# Increased Susceptibility to Ischemic Brain Injury in Neuroplastin 65-Deficient Mice Likely via Glutamate Excitotoxicity

**DOI:** 10.3389/fncel.2017.00110

**Published:** 2017-04-19

**Authors:** Yuhui Hu, Qin Zhan, Haibo Zhang, Xiaoqing Liu, Liang Huang, Huanhuan Li, Qionglan Yuan

**Affiliations:** ^1^Department of Neurology, Shanghai Tongji Hospital, Tongji University School of MedicineShanghai, China; ^2^Department of Anatomy, Jinggansan University School of MedicineJian, China; ^3^Department of Neurology, Seventh People's Hospital of Shanghai University of Traditional Chinese MedicineShanghai, China

**Keywords:** neuroplastin 65, stroke, ERK1/2 signaling, NMDA receptor, glutamate

## Abstract

Cell adhesion molecules (CAMs) are involved in synaptic plasticity and neuronal survival in the adult brain. Neuroplastin 65 (Np65), one member of the immunoglobulin superfamily of CAMs, is brain-specific and highly expressed in rodent forebrain. The roles of Np65 in synaptic plasticity have been confirmed, however, whether Np65 affects neuronal survival remains unknown. To address this gap, we generated, to our knowledge, the first Np65 knockout (KO) mice. By occluding middle cerebral artery to perform ischemic stroke model, we showed that Np65 KO mice exhibited more severe neurological deficits and larger infarction volume measured by TTC staining and more apoptotic cells confirmed by TUNEL staining compared to wild type (WT) mice. Besides, western blot analysis showed that the vesicular glutamate transporter-1(VGluT1), and N-Methyl D-Aspartate receptors, including NR1, NR2A, and NR2B were significantly increased in Np65 KO mice compared with WT mice. In contrast, vesicular gamma amino butyric acid transporter (VGAT) levels were unchanged in two genotypes after stroke. Additionally, phosphorylated-extracellular signal-regulated kinase 1/2 levels were significantly increased in Np65 KO mice compared with WT mice after stroke. Together, these results suggest that Np65 KO mice may be more susceptible to ischemic events in the brain.

## Introduction

Cell adhesion molecules (CAMs) are crucially involved in neuronal differentiation during development and support the maturation and maintenance of synapses in the adult brain. Neuroplastin (Np) is a glycoprotein belonging to the immunoglobulin (Ig) superfamily (IgSF) of CAMs (Langnaese et al., 1997). For alternative splicing of the transcript from a single gene, Np exists in two isoforms, Np55 and Np65 according to molecular weights. The isoforms can be distinguished solely by the presence of the Ig1 module specific to Np65 and absent in Np55 (Langnaese et al., 1997; Smalla et al., 2000). Of the two isoforms, Np65 is brain-specific, whereas Np55 is widely expressed in various tissues (Hill et al., 1988; Langnaese et al., 1997, 1998; Smalla et al., 2000). In the rodent brain, Np65 is predominantly located in forebrain such as the cortex, striatum and hippocampus (Hill et al., 1988; Langnaese et al., 1997, 1998; Smalla et al., 2000).

Accumulating evidence shows that Np65 mediates activity–dependent synaptic plasticity and neuritogenesis and neuronal plasticity (Smalla et al., 2000; Empson et al., 2006; Owczarek et al., 2010). For example, the recombinant ectodomain of Np65 and anti-Np antibodies inhibit long-term potentiation (LTP) in the CA1 region of the hippocampus (Smalla et al., 2000; Empson et al., 2006), probably via p38 MAPK-dependent destabilization and internalization of GluA1-containing glutamate receptors from the synaptic membrane (Empson et al., 2006). In addition, our previous studies have found that Np65 may be involved in modulation of neuritogenesis *in vitro* (Owczarek et al., 2011). More recently, we found that Np65 knock-out mice exhibit enhanced hippocampus-dependent learning and memory and anxiety-like behaviors (Amuti et al., 2016).

CAMs also play an important role in neuronal survival (Hulley et al., 1998; Ditlevsen et al., 2003, 2007). However, very limited information is available regarding roles of Np65 in neuronal survival, with exception to our observations that the peptides derived from NP Ig1 or Ig2 increase the survival of cerebellar granule neurons induced to undergo apoptosis (Owczarek et al., 2011), suggesting that Np65 interaction protects neurons against apoptosis *in vitro*.

Here, we performed ischemic stroke model by using Np65 KO mice to investigate the roles of Np65 in neuronal survival *in vivo*. We identified for the first time Np65 KO mice suffered more severe ischemic injury after stroke and indicate protective effects of Np65 in the brain.

## Materials and methods

### Np65 KO mice

Animal care and experimental protocols complied with National Institutes of Health Guide for the Care and Use of Laboratory Animals, were also approved by the Animal Study Committee at Tongji University School of Medicine. Generation and characterization of Np65 knock-out mice (KO) and their WT littermates were described in our lab (Amuti et al., 2016). In brief, C57BL/6J Np65 heterozygotes (Np65^+/−^) mice were backcrossed to C57BL/6J mice (Slac Laboratory Animal Center, Shanghai) for at least five generations and resulting offspring were subsequently bred to generate homozygous offspring. Genotyping of the offspring was performed by PCR of tail DNA extracts and by Western blot of Np65 (Amuti et al., 2016).

### Focal cerebral ischemia model

Focal cerebral ischemia model was performed by transiently occluding middle cerebral artery (MCA) as described (Yuan et al., 2007). In brief, Np65 KO mice and WT mice (2–4 months, 25–30 g) were anesthetized with 1% pentobarbital sodium in 0.9% NaCl. The right common carotid artery, external carotid artery, and internal carotid artery were exposed and a 10-0 monofilament nylon (10 mm long, Beijing Sunbio Biotech Co. Ltd.) was advanced from the common carotid artery into the lumen of the internal carotid artery until it blocked the origin of the MCA. Two hours after ischemia, the MCA ligature was removed and the animals were killed at 1 week after reperfusion. After surgery, mice exhibiting neurological deficits characterized by failure to extend the left forepaw were considered as successful models. The mice were allowed to survive for 1 week. All animals were maintained on a 12 h light-dark cycle in a temperature-controlled room with *ad libitum* access to water and food. All efforts were made to minimize pain and distress and the methods were carried out in accordance with the relevant guidelines.

### Neurological behavioral tests

Behaviors were tested at 7 days after stroke. Modified Neurological Severity Scale (mNSS) scores were used to evaluate the neurological function according to the previous methods (Yuan et al., 2007). The mNSS was a composite of motor, sensory, reflex, and balance tests. Neurological function was graded on a scale of 0–18 (normal, 0; maximal deficit score, 18). The higher the score, the more severe the injury was (seen in Table 1).

**Table 1:** **Modified Neurological Severity Score Points**.

**Motor tests**	
Raising rat by tail	3
Flexion of forelimb	1
Flexion of hindlimb	1
Head moved > 10° to vertical axis within 30 s	1
**Placing rat on floor (normal** = **0; maximum** = **3)**	3
Normal walk	0
Inability to walk straight	1
Circling toward paretic side	2
Falls down to paretic side	3
**Sensory tests**	2
Placing test (visual and tactile test)	1
Proprioceptive test (deep sensation, pushing paw against table edge to stimulate limb muscles)	1
**Beam balance tests (normal** = **0; maximum** = **6)**	6
Balances with steady posture	0
Grasps side of beam	1
Hugs beam and 1 limb falls down from beam	2
Hugs beam and 2 limbs fall down from beam, or spins on beam (>60 s)	3
Attempts to balance on beam but falls off (>20 s)	5
Falls off; no attempt to balance or hang on to beam (<20 s)	6
**Reflex absence and abnormal movements**	4
Pinna reflex (head shake when auditory meatus is touched)	1
Corneal reflex (eye blink when cornea is lightly touched with cotton)	1
Startle reflex (motor response to a brief noise from snapping a clipboard paper)	1
Seizures, myoclonus, myodystony	1
**Maximum points**	18

### Determination of lesion volume

Infarct volume was measured by 2, 3, 5-triphenyltetrazolium chloride (TTC, Sigma) staining as previously described (Horita et al., 2006). Np65 and WT mice (*n* = 4 per group) were killed by overdose of 1% pentobarbital sodium 7 days after reperfusion and brains were removed carefully and dissected into 6 coronal blocks (1.5 mm thick each). The fresh blocks were immersed in a 2% solution of TTC in saline at 37°C for 30 min and then were imaged by a digital camera (Canon, EOS 40D). The infarct volume was calculated indirectly by subtracting intact area of the ipsilateral hemisphere from the area of the contralateral hemisphere (Swanson et al., 1990; Yang et al., 2011). The results were expressed as percentage of lesion volume compared with contralateral hemisphere area, considering that as 100%.

### Brain tissue preparation

All mice (*n* = 4) were killed by overdose of 1% pentobarbital sodium at 7 days after stroke and transcardially perfused with PBS followed by 4% PFA. In addition, untreated WT and Np65 KO mice (*n* = 4) were considered as control mice. Brains were removed and post-fixed in 4% PFA for 10–16 h, and cryoprotected in 20% sucrose before sectioning with a cryostat (CM 1950, Leica, Heidelberger, Germany). Coronal sections (25 μM thick) were cut and processed for following staining.

### Nissl staining

Nissl staining was performed to evaluate neurons as described previously (Zhang et al., 2012; Chen et al., 2013). After washing with PBS, the sections were incubated with Nissl Staining Solution (Beyotime Institute of Biotechnology, Nanjing, China) for 20 min at room temperature. Sections were then dehydrated with ethanol and covered with Permount.

### TUNEL staining

Terminal deoxynucleotidyltransferase-mediated dUTP nick end labeling (TUNEL) staining was used to identify apoptotic cells under light microscope and was performed as described (Matsushita et al., 2000). *In situ* Cell Death Detection kit (Roche Molecular Biochemicals) was used. Briefly, after sections were treated with 3% H_2_O_2_ at room temperature for 15 min and then digested with proteinase K (20 μg/ml) at 37°C for 15 min, each section was incubated with 50 ml of TUNEL reaction mixture (5 ml of enzyme solution and 45 ml of label solution) at 37°C for 60 min, then followed with peroxidase at 37°C for 30 min with diaminobenzidineor for 3–5 min. Negative controls were performed with label solution instead of TUNEL reaction mixture. Sections were counterstained with hematoxylin, cleared and covered with Permount.

### Quantification analyses for staining

For semiquantitative measurements of Nissl and TUNEL staining, four animals per group were analyzed. Three slides with five intervals from sequential hippocampus and striatum sections were chosen in each mouse. Sections were imaged with a light microscopy (Nikon, ECLIPSE, E600, Tokyo, Japan) with three non-overlapping fields under a 200 × magnification. The number of neurons in the hippocampus and striatum (for Nissl staining) or the number of TUNEL-positive cells in the hippocampus and striatum was counted with Image Tool Software (Pro Plus v 6.0). All analyses were performed blinded to the genotype of the animals.

### Western blotting

At 7 days after MCAO, Np65 KO, and WT mice (*n* = 3) were sacrificed under deep anesthesia with pentobarbital sodium. In addition, untreated WT and Np65 KO mice (*n* = 3) were considered as control mice. Whole brain was immediately removed and right (ipsilateral) forebrain was collected and rapidly frozen in liquid nitrogen and then stored at −80°C until homogenization.

Frozen tissue was weighed, thawed, and homogenized. The samples were centrifuged at 14,000 rpm at 4°C for 20 min and the supernatant was collected. Protein concentrations were measured using a BCA Protein Assay Kit (Beyotime Institute of Biotechnology, Nanjing, China). Proteins were separated by SDS-PAGE and transferred to the nitrocellulose membranes. After blocking with 10% skim milk, the blots were incubated overnight at 4°C with primary antibodies: rabbit anti-Vesicular GABA transporter (VGAT, 1:500), rabbit anti-vesicular glutamate transporter-1 (VGLuT1), phospho-ERK1/2 (Thr 202/Tyr 204, p-ERK1/2) (1:000, Cell Signaling Technology), ERK1/2 (1:1,000, Cell Signaling Technology), mouse anti-GAPDH (1:1,000, Santa Cruz Biotechnology). Subsequently, the membrane was incubated with secondary antibodies against the primary antibody for 1 h at room temperature, and the labeled proteins were detected by using the Bio-Rad system and quantified through Quantity One analysis. The final results were expressed as a ratio of the expression of the protein of interest to that of GAPDH.

### Data analysis

Statistical analysis was performed with SPSS13.0 for Windows. All data were expressed as mean ± SEM. Multiple comparisons were performed by using one-way ANOVA followed by Tukey's *post hoc* test for multiple pair-wise examinations *in vivo* experiments. The difference was considered significant if *p* < 0.05.

## Results

### More severe neurological deficits and larger infarct volume in Np65 KO mice after stroke

**Figure 1:**
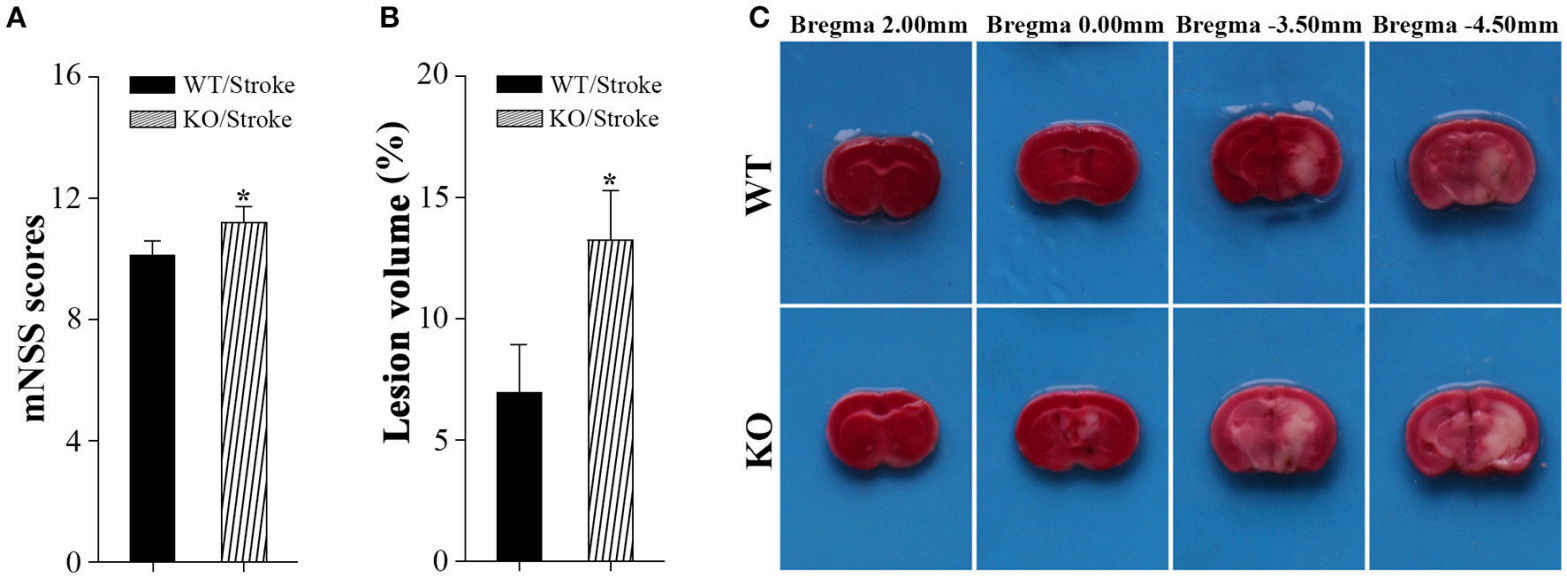
**Np65 deficiency exacerbates neurological deficits and increases infarct volume in mice after stroke**. **(A)** Neurological function was determined by modified neurological severity scores (mNSS), summarizing the results of motor, sensory, reflex, and balance tests. Np65 KO mice exhibited higher scores at 7 days after stroke. *N* = 8. ^*^p < 0.05; **(B)** Quantitative image analysis of infarct volume. Np65 deficiency increases infarct volume in mice at 7 days after stroke. **(C)** Representative brain slices were stained by TTC. Normal brain tissue was red and infarct tissue was white; *N* = 4. ^*^p < 0.05.

Np65 gene deletion did not affect locomotor activity of mice (Amuti et al., 2016). Before stroke, both type mice exhibited normal motor movement, sensory, reflex, and balance, the scores were 0. At 7 days after stroke, neurological performance was evaluated by modified neurological severity score (Yang et al., 2011). The higher scores indicate the more severe injury (Yang et al., 2011). As shown in Figure 1, higher scores in Np65 KO mice were observed compared to WT mice after stroke, suggesting that Np65 deficiency exacerbates neurological deficits in mice after stroke.

In addition, infarct volume, another index suggesting the extent of injury, was calculated by TTC staining at 7 days after stroke. The ischemic volume was indirectly calculated by subtracting intact area of the ipsilateral hemisphere from the area of the contralateral hemisphere (Swanson et al., 1990). The remaining normal brain tissue was typically stained with TTC and appeared red color, and infarct tissue showed no or reduced staining and appeared white color (Bederson et al., 1986). As shown in Figure 1C, the contralateral hemispheres and remaining normal tissues in the ipsilateral hemisphere appeared red color. Meanwhile, ischemic regions in ipsilateral hemisphere appeared white color and were primarily located in hippocampus and striatum. Infarct volume was significantly increased in Np65 KO mice compared to WT mice (Figure 1C). These results indicate that Np65 deficiency increases infarct volume in mice after stroke.

### Fewer neurons in the ipsilateral hemisphere in Np65 KO mice after stroke

**Figure 2:**
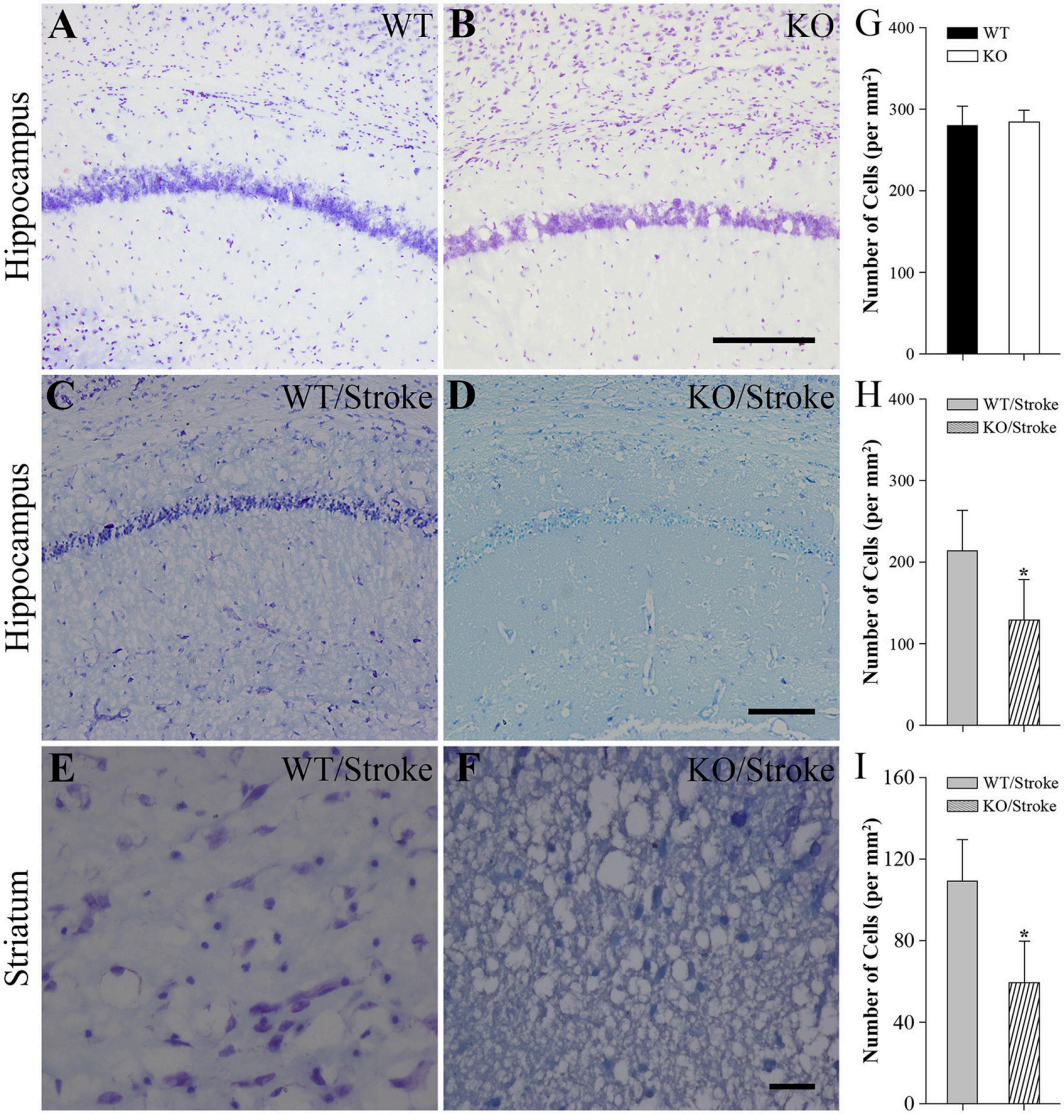
**Nissl staining showed fewer neurons of hippocampus and striatum in Np65 KO mice after stroke**. **(A–F)** Representative Nissl staining showed neurons of hippocampus **(A–D)** and striatum **(E,F)** in WT and Np65 KO mice in normal **(A,B)** and after stroke **(C–F)**. Scale bar = 200 μm **(A–D)** or 50 μm **(E,F)**; **(G–I)** Histogram showed that the number of neurons of hippocampus was no different between WT and Np65 KO mice **(G)**, but a significant decrease in the ischemic hippocampus **(H)** and striatum **(I)** was observed in Np65 KO mice after stroke. *N* = 4. ^*^p < 0.05.

Nissl staining was used to observe the histologic features in mice after stroke. In untreated normal WT and Np65 KO mice, no abnormal neurons were observed and the amounts of neurons in cortex and hippocampus did not differ (Figure 2). In contrast, at 7 days after stroke, the majority of neurons in hippocampus and striatum disappeared and some neurons shrunk with pyknotic nuclei (Figure 2). The numbers of remaining neurons in ischemic hippocampus and striatum were measured and we found that the numbers of remaining neurons were significantly decreased in Np65 KO mice compared to WT mice after stroke for 7 days. These results suggest that Np65 deficiency leads to a more severe injury in mice after stroke.

### More apoptotic cells in Np65 KO mice after stroke

**Figure 3:**
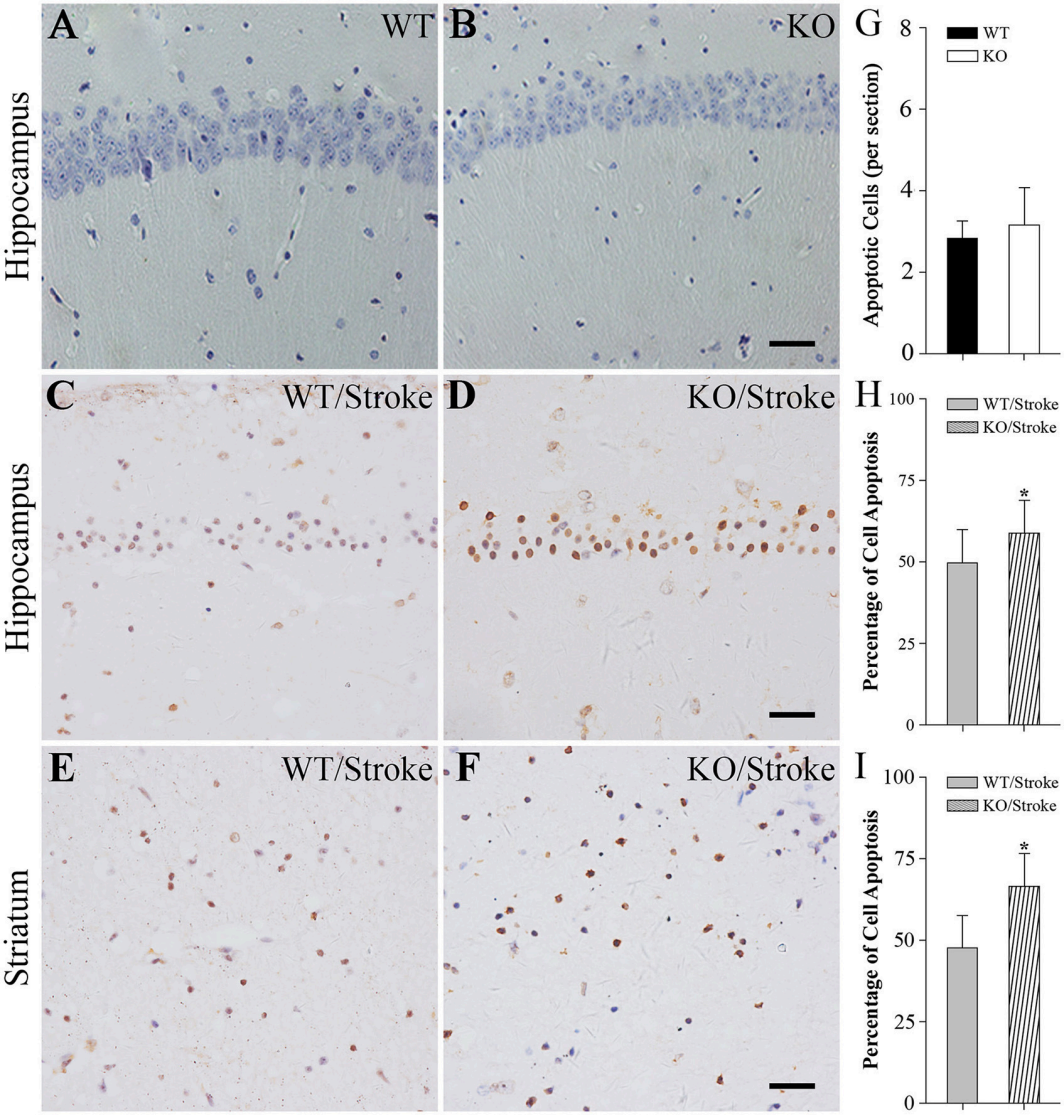
**TUNEL staining showed more apoptotic cells in Np65 KO mice after stroke. (A–F)** Representative TUNEL staining showed that apoptotic cells (↑) in hippocampus **(A–D)** and striatum **(E,F)** in Np65 KO and WT mice in normal **(A,B)** and after stroke **(C–F)**. Scale bar = 25 μm; **(G–I)** Histogram showed that the apoptotic cells of hippocampus was no different between WT and Np65 KO mice **(G)**, but a significant increase in the ischemic hippocampus **(H)** and striatum **(I)** was observed in Np65 KO mice after stroke. *N* = 4. ^*^p < 0.05.

In addition, the apoptotic cells were measured by using TUNEL staining. In normal Np65 KO and WT mice, very fewer of TUNEL-positive cells were observed and the number of TUNEL-positive cells did not differ (Figure 3). In contrast, many TUNEL-positive cells were present in the hippocampus and striatum at 7 days after stroke. The numbers of apoptotic cells in hippocampus and striatum were significantly increased in Np65 KO mice compared to WT mice (Figure 3).

### Altered ERK1/2 signal in Np65 KO mice after stroke

**Figure 4:**
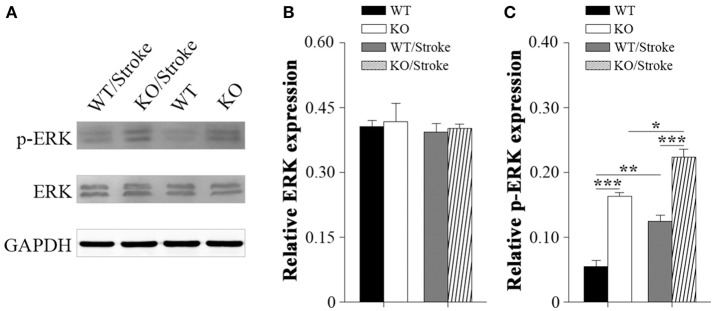
**Increased p-ERK1/2 signaling in Np65 KO mice after stroke**. **(A)** Representative immunoblot bands of ERK1/2, phosphorylated-ERK1/2 (p-ERK1/2) from ipsilateral forebrain in Np65 KO and WT mice in normal and after stroke; **(B–C)** Quantification of immunoreactivity bands normalized against GAPDH showed that the p-ERK1/2 levels were significantly increased in Np65 KO mice compared to WT mice but the total ERK1/2 levels were unchanged. After stroke, p-ERK1/2 levels were significantly increased in two genotypes. Moreover, far higher p-ERK levels were observed in Np65 KO mice compared to WT mice after stroke. *N* = 3. ^*^p < 0.05; ^**^p < 0.01, ^***^p < 0.001.

Mitogen-activated protein kinases (MAPK) are involved in many physiological and pathological processes. The MAPK family includes extracellular signal-regulated kinase 1/2(ERK1/2), c-Jun N-terminal kinase (JNK), and p38 MAPK (Roux and Blenis, 2004). ERK1/2 signaling has been implicated in synaptic plasticity and memory formation as well as survival (Sweatt, 2004). To explore the underlying mechanisms that Np65 KO mice are susceptibility to ischemia, the ERK1/2 signaling was studied. As shown in Figure 4, p-ERK1/2 levels were significantly increased in Np65 KO mice compared to WT mice although the total ERK1/2 levels were unchanged. At 7 days after stroke, p-ERK1/2 levels were significantly increased in two genotypes. Moreover, far higher p-ERK levels were observed in Np65 KO mice compared to WT mice after stroke. These results suggest that p-ERK1/2 levels are significantly increased in Np65 KO mice after stroke.

### Increased glutamate and NMDAR in Np65 KO mice after stroke

**Figure 5:**
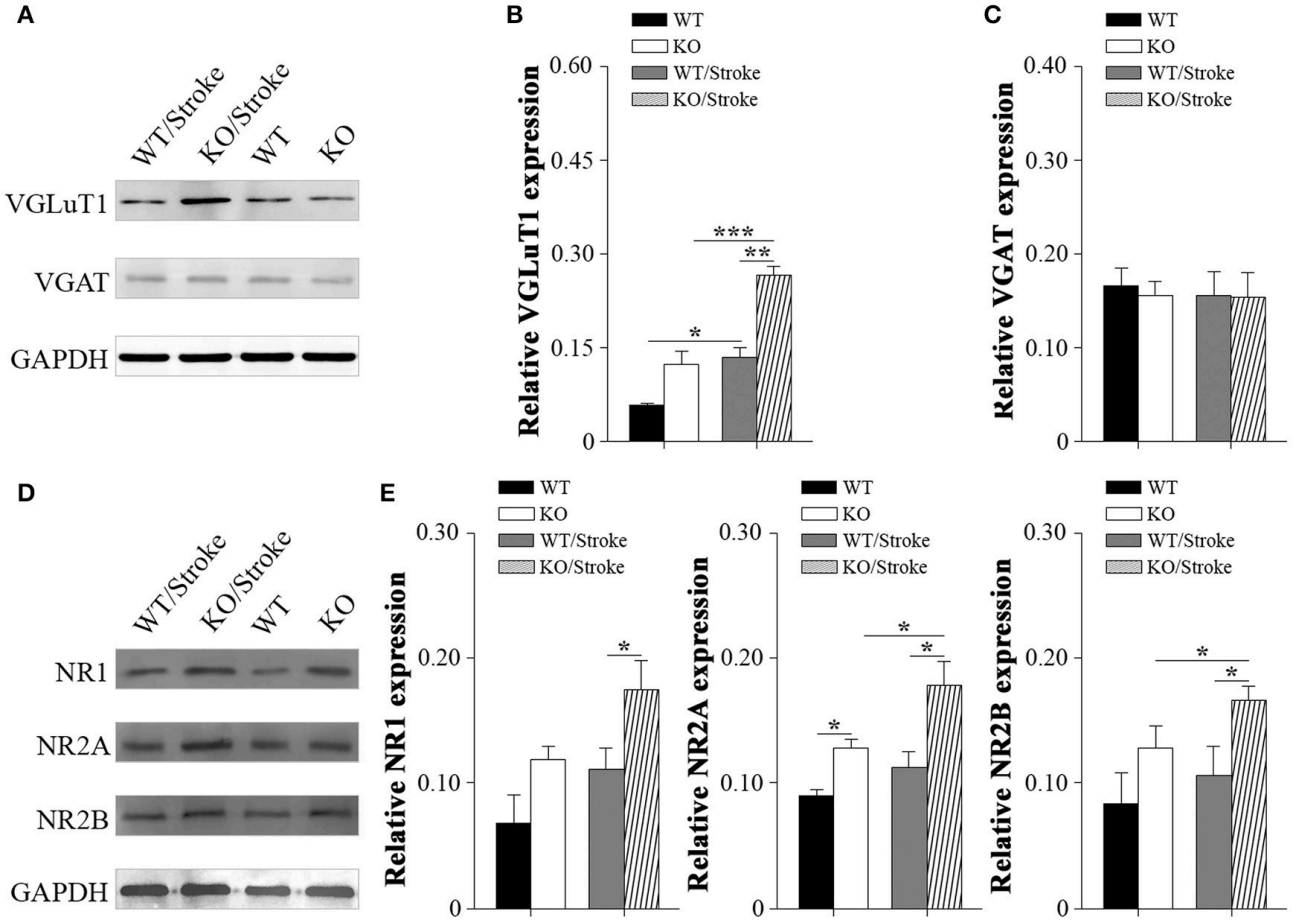
**Altered neurotransmitters and NMDA receptors in Np65 KO mice after stroke**. **(A)** Representative immunoblot bands of VGLuT1 and VGAT from ipsilateral forebrain of mice in normal and after stroke; **(B–C)** Quantification of immunoreactivity bands normalized against GAPDH showed the increased VGlut1 levels and unchanged VGAT levels in Np65 KO mice compared to WT mice. At 7 days after stroke, VGLuT1 expression levels were significantly increased in two genotypes, and much higher levels of VGlut1 appeared in Np65 KO mice. However, VGAT protein levels were unchanged in mice after stroke. *N* = 3, ^*^p < 0.05, ^**^p < 0.01, ^***^p < 0.001; **(D)** Representative immunoblot bands of NMDAR subunits from ipsilateral forebrain of Np65 KO and WT mice in normal and after stroke; **(E)** Quantification of immunoreactivity bands normalized against GAPDH showed the expressions of NR2A were apparently higher in Np65 KO mice compared to WT mice. At 7 days after stroke, the NR2A and NR2B expressions were significantly elevated in Np65 KO mice but not in WT mice. Notably, NR1, NR2A, and NR2B expressions were significantly elevated in Np65 KO mice compared to WT mice after stroke. *N* = 3, ^*^p < 0.05.

Excitotoxicity has been well accepted to be one of the principal pathogenesis of ischemic stroke. Therefore, inhibitory/excitatory neurotransmitters were explored in mice after stroke. Vesicular gamma amino butyric acid transporter (VGAT), an indirect marker for inhibitory GABA levels, and vesicular glutamate transporter-1 (VGluT1), an indirect marker for glutamate levels, were measured in forebrain by western blot analysis. The results revealed that VGlut1 levels in Np65 KO mice were significantly increased compared to WT mice. In contrast, VGAT protein levels were unchanged in two genotypes. At 7 days after Stroke, VGlut1 expression levels were significantly increased, and much higher levels of VGlut1 in Np65 KO mice were observed in comparison with WT mice (Figures 5A–C). However, VGAT protein levels were unchanged in mice after stroke. In addition, N-Methyl D-Aspartate receptor (NMDAR), a high-affinity of glutamate, was evaluated. The NMDAR is a heterotetrameric complex composed of two obligatory NR1 subunits and two NR2 subunits. The results showed that the expressions of NR2A were apparently higher in Np65 KO mice compared to WT mice. At 7 days after stroke, the NR2A and NR2B expressions were significantly elevated in Np65 KO mice not in WT mice. Notably, NR1, NR2A, and NR2B expressions were significantly elevated in Np65 KO mice compared to WT mice after stroke (Figures 5D–E). These results suggest that elevated glutamate and enhanced NMDAR levels in Np65 KO mice may contribute, at least partially, to more severe damage after stroke.

## Discussion

In the present study, we present evidence that Np65, similar to other CAMs, is also involved in neuronal survival *in vivo*. We evaluated the role of Np65 in neuronal survival by using Np65 KO mice, and found that Np65 KO mice exposed to ischemic stroke exhibit more severe neurological deficits and larger infarct volume and more apoptotic cells after stroke. Besides, the VGluT1 and NMDA receptors, including NR1 and NR2A, NR2B, p-ERK1/2 levels are significantly increased in Np65 KO mice compared with WT mice after stroke. Together, these results suggest that Np65 KO mice may be susceptible to ischemic events in the brain.

Although previous findings document that CAMs play crucial roles in neural development and are also critically involved in neurite outgrowth and synaptic plasticity in the adult nervous system (Hulley et al., 1998; Kiryushko et al., 2006), the roles of Ig SF CAMs in survival of neurons have only been recognized in different pathological settings (Hulley et al., 1998; Gary and Mattson, 2001; Ditlevsen et al., 2003, 2007). For example, the neural cell adhesion molecule (NCAM) activation, in addition to leading to neurite extension, also leads to an increased survival of primary dopaminergic neurons induced by 6-OHDA to undergo apoptosis (Ditlevsen et al., 2007). L1 neural cell adhesion molecule, another one of IgSF CAMs, promotes survival of fetal dopaminergic neurons by using L1 antibody *in vitro* (Hulley et al., 1998). Integrin exhibits increased resistance to glutamate-induced apoptosis (Gary and Mattson, 2001). Our previous studies have shown that Np65 gene deletion does not affect locomotor activity and brain framework of organization in adult mice (Amuti et al., 2016). In addition, we showed that peptides derived from NP Ig1 or Ig2 significantly increased the survival of primary cerebellar granule neurons induced to undergo apoptosis by low KCl in the media (Owczarek et al., 2011), suggesting that Np65 protects neurons against apoptosis *in vitro*. To further explore the possible roles of Np65 in neuronal survival *in vivo*, we made a stroke model using Np65 KO mice to observe the ischemic damage and neurological behaviors. We found that Np65 KO mice showed more severe neurological deficits, larger infarct and more apoptotic cells compared to WT mice after stroke. To our knowledge, it is the first time to demonstrate that Np 65 KO mice show susceptibility to cerebral ischemia.

Glutamate excitotoxicity is believed to be one of the early events in propagating neurological damage in stroke (Choi, 1992; Iadecola et al., 2001). MK801, a NMDA receptor blocker, has been shown to alleviate ischemic damage in rodent after stroke (Buchan et al., 1992; Gerriets et al., 2003). In our present study, Np65 KO mice were more susceptible to ischemia induced by MCAO than WT mice. To explore the underlying mechanisms, glutamate and NMDA receptors were investigated by western blot analysis. VGluT1, an indirect marker for glutamate levels, and NMDA receptors, including NR1 and NR2A and NR2B, were observed. We found that the expressions of NR2A were significantly increased in Np 65KO mice compared to WT mice. Whether these increases affect brain function needs to be investigated. At 1 week after stroke, VGluT1 and NMDA receptors including NR1 and NR2A and NR2B, were significantly increased in Np65 KO mice compared with WT mice. Contrary to these results, the VGAT, an indirect marker for inhibitory neurotransmitter GABA, was unchanged. Previous studies have confirmed that NP65 trans-homophilic binding activates p38MAPK, which in turn down-regulates surface GluR1 glutamate receptors by internalization in cultured hippocampal neurons (Empson et al., 2006). Moreover, it has shown that Np65 co-localizes with a1 and a2, but not a3 subunits at GABAergic synapses and a5 subunits at extra synaptic sites in cultures (Sarto-Jackson et al., 2012). These results have shown that Np65 affects composition of PSDs, especially transmitter receptors and may involve excitatory and inhibitory synaptic transmission. More recently, Carrott et al. reported that Np65-decicient mice show abnormal synaptogenesis in inner hair cells and causes profound hearing loss (Carrott et al., 2016). Combined our results with these previous studies, it is speculated that the highly increased glutamate and NMDA receptors might render excitotoxicity in Np65 KO mice after stroke and lead to excitotoxic neuronal death. How Np65 deficiency affects the glutamate and its receptors is hitherto unknown in mice after stroke and needs to be further investigated in our next step.

Extracellular signal–regulated kinase 1/2 (ERK1/2), one major member of mitogen-activated protein kinases (MAPK) family, has been implicated in crucial roles in synaptic plasticity and hippocampus-dependent memory (Sweatt, 2004) as well as in cerebral ischemia (Sawe et al., 2008). Our previous study has confirmed that Np65-induced neurite outgrowth involves the activation of ERK1/2 (Owczarek et al., 2011; Beesley et al., 2014). In present study, the ERK1/2 signaling was observed in the ischemic forebrain homogenate at 7 days after stroke. There were no significant differences in total ERK levels between these groups. p-ERK1/2 levels were increased in Np65 KO mice compared to WT mice, which might be related to improved learning and memory in Np65 KO mice (Amuti et al., 2016). Importantly, at 7 days after stroke, a higher increase in p-ERK1/2 levels was observed in Np65 KO mice compared to WT mice. Whether an increase in p-ERK1/2 is protective or detrimental is highly debatable (Sawe et al., 2008). For instance, ERK activity may exaggerate inflammation by up-regulating interleukin 1β (IL-1β), thus leading to necrosis (Wang et al., 2004); it may also block apoptosis by enhancing the level of the antiapoptotic protein Bcl-2 or blocking the proapoptotic protein Bad (Purcell et al., 2003; Roux and Blenis, 2004). In our ischemic settings, Np65 KO mice showed more severe neuronal loss in striatum and hippocampus at 7 days after stroke. Therefore, an increase in p-ERK1/2 might be partially associated with exacerbated damage in Np65 KO mice after stroke. Because p-ERK is differentially expressed in discrete regions of ischemic brains (Hu et al., 2000; Ferrer and Planas, 2003; Zablocka et al., 2003), the limitation of this study is a lack of p-ERK1/2 immunohistological staining to show distribution of p-ERK1/2 positive cells, which helps to understand effects of p-ERK1/2 in this paradigm.

In conclusion, we demonstrated for the first time that Np65 KO mice exhibit more severe neurological deficits and larger infarct volume and more apoptotic cells after ischemic stroke than WT mice. The increased VGluT1 and NMDA receptors and p-ERK1/ 2 may contribute to the increased susceptibility to ischemia in Np65KO mice.

## Author contributions

QY designed the work and wrote the manuscript; YH, LH, HZ, and QZ performed the experiments; HL analyzed the data; XL revised the draft. All authors approved the submission.

### Conflict of interest statement

The authors declare that the research was conducted in the absence of any commercial or financial relationships that could be construed as a potential conflict of interest.
